# Research on agroforestry systems and biodiversity conservation: what can we conclude so far and what should we improve?

**DOI:** 10.1186/s12862-022-01977-z

**Published:** 2022-03-03

**Authors:** Sébastien Boinot, Karim Barkaoui, Delphine Mézière, Pierre-Eric Lauri, Jean-Pierre Sarthou, Audrey Alignier

**Affiliations:** 1 UMR 0980 BAGAP, INRAE-Institut Agro-ESA, 65 rue de St Brieuc CS 84215, 35042 Rennes Cedex, France; 2 ABSys, Univ Montpellier, CIHEAM­IAMM, CIRAD, INRAE, Institut Agro, Montpellier, France; 3 CIRAD, UMR ABSys, Montpellier, France; 4 University of Toulouse, INRAE, INPT-ENSAT, UMR AGIR, 31326 Castanet-Tolosan, France; 5 LTSER « Zone Atelier Armorique », 35042 Rennes, France

**Keywords:** Alley cropping, Control plot, Experimental design, Farming practices, Diversity indices, Hedgerow, Meta-analysis

## Abstract

Through a meta-analysis, Mupepele et al. (BMC Ecol Evol 21:1–193, 2021) assessed the effects of European agroforestry systems on biodiversity, estimated by species richness or species diversity. They showed that the effects of silvoarable and silvopastoral systems depend on the systems they are compared to and the taxa studied. Further, they found that only silvoarable systems increased species richness or diversity, compared to cropland. The authors conclude that agroforestry systems have weak effects on biodiversity and that landscape context or land-use history are probably more important than the practice of agroforestry in itself. However, we draw attention to important shortcomings in this meta-analysis, which downplay the potential of agroforestry for biodiversity conservation in agricultural landscapes. We hope that the meta-analysis by Mupepele et al. (BMC Ecol Evol 21:1–193, 2021), and our comments, will contribute to improving the quality of research on agroforestry systems and biodiversity conservation.

## Hedgerow systems are widespread agroforestry systems in Europe

In their meta-analysis, Mupepele et al. [1] defined agroforestry as a “collective name for diverse land-use systems integrating tree husbandry with livestock or arable cultivation”. Fields bordered by riparian fields margins and hedgerows were excluded as “they are not really under silvicultural use”. However, hedgerows are widespread traditional agroforestry practices and were part of the AGFORWARD European research project (AGroFORestry that Will Advance Rural Development) [2]. We emphasize that farmers can use hedgerows for wood production and include them in their farm management. For instance, based on a recent survey [3], 20% of farmers plant hedgerows in Brittany. Among them, 70% valorize the wood resulting from the maintenance of hedgerows (e.g., firewood, timber wood, wood chips, ramial-chipped wood). Hedged farmland (or bocage) are agroforestry systems with high potential to enhance the diversity of many taxa, including plants, invertebrates, birds, and mammals [4, 5]. In European agricultural landscapes, hedgerows are particularly interesting for biodiversity conservation as they are relatively stable habitats, offering refugia and trophic resources to many living organisms [6] and references therein). Compared to modern agroforestry systems (e.g., alley cropping), there is a large body of work on hedgerows and their management, which should be accounted for in a synthesis work on agroforestry. This could only improve our knowledge of ecological processes in agroforestry systems and their impacts on biodiversity conservation.

## Inadequate controls to assess the effects of agroforestry systems

We carefully read the protocols of studies comparing agroforestry systems with arable crops or open pastures, as our primary interest is the benefit of agroforestry systems in agricultural landscapes. We found that at least 11 out of 28 studies included in the meta-analysis by Mupepele et al. [1] did not have adequate control sites. Many studies were designed to assess the distribution of taxa within agroforestry systems [7–11], not to assess the effect of agroforestry relative to other cropping systems. For instance, Boinot et al. [7] assessed the distribution of overwintering invertebrates in alley cropping systems, comparing assemblages in crop alleys vs. understory vegetation strips. Mupepele et al. then used crop alleys as cropland controls in their meta-analysis. The authors stated that “observational studies were included if a good proxy for a control site was available. This was the case for studies about species groups with limited mobility (e.g. plants or Collembola) looking at distance gradients”. This argument is not valid because species with limited mobility can still be affected by the agroforestry microclimate [12, 13], or by biotic interactions with more mobile species. First, increased environmental heterogeneity in agroforestry systems could favor species coexistence and diversification [14]. Second, studies found that agroforestry can promote predators and seed dispersers (e.g., [15–17], and pollinators [18] and references therein). Predator partitioning, the presence of dispersal vectors, and complex plant-pollinator networks are essential for maintaining plant diversity [19–21]. It should also be emphasized that herbaceous plant species can have high dispersal abilities [22, 23]. Besides, the authors included studies with controls that are located close to the tree rows, for instance, at distances varying between 18 and 55 m [24], between 20 and 30 m [25], or even less than 10 m [26]. These distances are far below the dispersal range of the taxa surveyed in these studies, including airborne arthropods caught with pan traps and sticky cards or ground-dwelling arthropods such as carabids and millipedes [27, 28].

Beyond inadequate controls, this meta-analysis includes a mixture of studies with different approaches. Some evaluate agroforestry systems with low-intensity management as an alternative to arable crops or open pastures with high-intensity management (e.g., [29, 30], whereas others disentangle the effect of agroforestry systems per se (i.e., the benefit of trees and spontaneous vegetation) from the effect of management intensity (e.g., [7, 31]). Besides, researchers rarely described the farming practices in agroforestry vs. control plots. The lack of a systemic approach hinders synthesis efforts such as those provided by the authors of this meta-analysis, and makes the results difficult to interpret. Overall, if the objective is to assess the effect of agroforestry per se, cropland controls should meet the following criteria to avoid confounding factors: (i) similar farming practices to that occurring in agroforestry systems (e.g., crop type, management intensity), (ii) location in the same landscape context, (iii) sufficient distance between agroforestry and cropland samples to minimize edge effects (e.g., microclimate, spillover). In all cases, farming practices should be reported as accurately as possible.

## Biodiversity goes beyond species counts

In the Discussion, the authors mention that “our conclusions are largely based on species richness comparison; communities may well differ in their composition beyond richness (compare e.g. [12, 25, 32]). For conservation decisions, variables such as the occurrence of rare and endangered species may be additionally relevant”. The fact that the authors draw conclusions about the effect of agroforestry systems on “biodiversity” is therefore highly misleading. As mentioned, they only considered one facet of biodiversity, species richness, which is an insufficient measure to characterize conservation areas or to describe the response of biodiversity to anthropogenic disturbances [33]. This meta-analysis does not provide information on abundance or species and functional composition, which may be severely affected. In the face of anthropogenic disturbances, the most profound changes in biodiversity are a high turnover of species and a reduction in functional diversity, whereas shifts in species richness show mixed patterns [34, 35]. Biotic homogenization can explain these results. Widespread generalist, fast-growing or invasive taxa tend to replace localized specialist, slow-growing, or threatened taxa, as they can cope or even thrive with anthropogenic disturbances [36, 37]. Such detrimental species turnover can occur without any change in species richness, or it can even lead to a punctual increase in species richness if colonization occurs more rapidly than extinctions.

We found that 22 out of 28 studies revealed effects of agroforestry systems on abundance (e.g., [31, 38–41], species composition (e.g., [40, 42–45], or ecological/functional groups (e.g., [38, 39, 41, 46, 47]. For instance, Varah [31] found that butterflies, which are well-known indicator organisms, were about three times more diverse and three times more abundant in agroforestry systems. Boinot et al. [38] showed that alley cropping systems are refugia for plant species that are sensitive to agricultural disturbances. Gallé et al. [43] identified new species for the Romanian spider fauna under the canopy of scattered trees in wood pastures, along with species of high conservation status. Rösch et al. [40] found higher bird species richness, beta diversity, and density of breeding pairs in wood pastures, along with the presence of target species for conservation, as opposed to open pastures. Therefore, the authors of this meta-analysis cannot put forward that “overall, there was no benefit of agroforestry to biodiversity” or that the effects on biodiversity are “of small magnitude”, when they only compared numbers of species.

## Too early for a meta-analysis on alley cropping agroforestry systems

It is too early to conclude on the effect of alley cropping agroforestry systems on biodiversity conservation. Results of studies are just beginning to emerge (e.g. [15, 48–51]). Only eight studies included in this meta-analysis involved alley cropping systems. Among them, most systems were still very young. Based on studies with adequate control sites, the mean age of alley cropping systems was 8.5 years (± 7.9). Thus, the results apply to systems in transition rather than to mature alley cropping systems. We can expect immigration credit with the development of trees [52], which could enhance biodiversity in older agroforestry systems. However, temporal studies are lacking. In this context, “before-after control-impact” designs should be promising [53].

Further, many studies used in this meta-analysis were not designed to assess the effect of alley cropping systems on biodiversity but were restricted to taxa of interest for agricultural production, such as crop pests or their natural enemies. We question the relevance of gathering such disparate studies in a meta-analysis, giving a false impression of exhaustive sampling. Indeed, the results of such studies are unlikely to reflect the response of biodiversity to a given system, as opposed to studies encompassing many taxa (e.g., [54]. Besides, out of the eight studies, five focused on arthropods, two on plants, and one on earthworms. Information on birds, mammals, reptiles, and amphibians is missing. Further studies are needed to assess the effect of temperate agroforestry systems on these taxa, which generally depend on the presence of semi-natural habitats in the surroundings [17, 55, 56].

Finally, in most studies, protocols were not designed to sample the full range of habitats that make up agroforestry systems. Often, this is explained by the identity of surveyed taxa and associated objectives (e.g., assessing pest pressure or biological control in crop alleys). In alley cropping systems, tree rows and associated vegetation strips were not always sampled, whereas they are crucial for biodiversity conservation. Such habitats provide refugia from agricultural disturbances (e.g., tillage, pesticide, harvest) and trophic resources [7, 57]), and probably serve as ecological corridors or stepping stones for species that do not venture into cropland. Notably, only one study sampled arthropods directly on trees [26]. In addition, non-selective herbicide treatments prevented the growth of herbaceous flora in some cases [25, 58]. Therefore, this meta-analysis most likely underestimates the positive effects of alley cropping systems on species diversity.

## Still plenty of room for improvement: the importance of local practices

As suggested by the authors, contrasted effects of agroforestry systems could be due to the landscape context or land-use history, which are rarely reported. We emphasize that many local factors could also affect biodiversity in agroforestry systems (and should be reported as well), such as (i) the density and diversity of tree, shrub, and herbaceous layers, (ii) their age, management and spatial configuration, and (iii) farming practices (e.g., tillage management, pesticide treatment, grazing intensity, fertilization, crop rotation, cover cropping). Management of semi-natural habitats such as hedgerows strongly affects biodiversity [59]. Tree diversity is a key driver of biodiversity in forests [60], and the same could hold in agroforestry systems [e.g. 61 in the tropics]. Further, intensive farming practices in cropped areas can undermine the potential of adjacent semi-natural habitats for biodiversity conservation [32, 62]. Therefore, the current performance of agroforestry systems is probably far from optimal, but studies on the effects of management at both local and landscape scales are lacking.

## Conclusion

Despite the strong enthusiasm of the agroforestry research community, a recent systematic review of agroforestry experiments in the Global South concludes that rigorous evidence on the effects of agroforestry on agricultural productivity, ecosystem services, and human well-being remains extremely limited [63]. Similarly, through their meta-analysis, Mupepele et al. [1] point out that we should not take for granted the positive effects of agroforestry on biodiversity. We add that we should not prematurely underestimate the potential of agroforestry either. There are reasons to be enthusiastic because many studies revealed tremendous impacts of agroforestry systems on the abundance, evenness, and composition of communities, which are all important facets of biodiversity. Future research on biodiversity conservation in agroforestry systems should (i) continue to go beyond species diversity by considering abundance and species, functional or genetic composition, (ii) encompass more taxa (e.g., micro-organisms, mammals, reptiles, and amphibians that are missing in this meta-analysis, (iii) assess the effects of local factors such as habitat management, spatial configuration, and farming practices, on which farmers can directly act, and (iv) assess the influence of land-use history and landscape context on agroforestry performance. To improve the quality of agroforestry research, we must take care developing rigorous experimental designs with adequate controls. Patience is also necessary; we have to wait for agroforestry systems to grow before jumping to meta-analyses and hasty conclusions.

## Data Availability

Not applicable.
